# Effect of postnatal environmental enrichment on LTP induction in the CA1 area of hippocampus of prenatally traffic noise-stressed female rats

**DOI:** 10.3934/Neuroscience.2023021

**Published:** 2023-10-20

**Authors:** Fatemeh Aghighi, Mahmoud Salami, Sayyed Alireza Talaei

**Affiliations:** Physiology Research Center, Institute for Basic Sciences, Kashan University of Medical Sciences, Kashan, I. R. Iran

**Keywords:** environmental enrichment, prenatal exposure, synaptic plasticity, rats

## Abstract

Early-life stress negatively alters mammalian brain programming. Environmental enrichment (EE) has beneficial effects on brain structure and function. This study aimed to evaluate the effects of postnatal environmental enrichment on long-term potentiation (LTP) induction in the hippocampal CA1 area of prenatally stressed female rats. The pregnant Wistar rats were housed in a standard animal room and exposed to traffic noise stress 2 hours/day during the third week of pregnancy. Their offspring either remained intact (ST) or received enrichment (SE) for a month starting from postnatal day 21. The control groups either remained intact (CO) or received enrichment (CE). Basic field excitatory post-synaptic potentials (fEPSPs) were recorded in the CA1 area; then, LTP was induced by high-frequency stimulation. Finally, the serum levels of corticosterone were measured. Our results showed that while the prenatal noise stress decreased the baseline responses of the ST rats when compared to the control rats (P < 0.001), the postnatal EE increased the fEPSPs of both the CE and SE animals when compared to the respective controls. Additionally, high-frequency stimulation (HFS) induced LTP in the fEPSPs of the CO rats (P < 0.001) and failed to induce LTP in the fEPSPs of the ST animals. The enriched condition caused increased potentiation of post-HFS responses in the controls (P < 0.001) and restored the disrupted synaptic plasticity of the CA1 area in the prenatally stressed rats. Likewise, the postnatal EE decreased the elevated serum corticosterone of prenatally stressed offspring (P < 0.001). In conclusion, the postnatal EE restored the stress induced impairment of synaptic plasticity in rats' female offspring.

## 1. Introduction

There is evidence that the intrauterine/fetal environment has long-term aftereffects on infant development [1]. Childhood is one of the sensitive periods in an individual's development. Exposure to stress during this period has a “programmed” effect on the structure and function of the central nervous system [2]. Studies have shown the effects of prenatal stress (PS) on the neuronal and synaptic development of several brain regions such as the neocortex, amygdala, hippocampus, and hypothalamus [3].

Acute or chronic fetal stress exposure induces hypothalamic-pituitary-adrenal (HPA) axis dysfunction and increased glucocorticoid secretion in animals [4]. In addition, maternal stress hormones such as adrenal steroids, catecholamines, and CRH reach the fetal brain and alter fetal neuronal structure and function [5]. Additionally, prenatal stress through the contraction of the placental artery due to activation of the mother's sympathetic nervous system reduces the supply of essential nutrients and oxygen, thereby negatively affecting the health of the fetus [6]. As we know, the hippocampus is very vulnerable to stress and plays a crucial role in spatial learning and memory [7]. It has been reported that prenatal stress impairs the spatial learning and memory of rats in a Morris water maze [8]. Theoretically, two types of hippocampal synaptic plasticity, long-term potentiation (LTP) and long-term depression (LTD), have been accepted as key mechanisms of certain types of learning and memory formation [9]. A previous study reported that prenatal stress impaired LTP but facilitated LTD within the CA1 area of the young rats' hippocampus [10].

In addition to understanding the harmful effects of prenatal stress on cognition, there is now considerable interest in developing a novel strategy to ameliorate these deficits. One such approach is utilizing environmental enrichment (EE) [11]. EE is a non-invasive therapy that creates strong changes in neuronal structure and behavior [12]. Within the central nervous system, EE enhances neurogenesis [13], synaptic plasticity [14], glutamate level [15], as well as decreases gamma-aminobutyric acid (GABA) [16]. At the molecular level, studies have demonstrated that exposure to EE alters various plasticity-related molecules such as synaptophysin [17], CaMKII, CREB [18], as well as N-methyl-D-aspartate (NMDA) receptor subunits GluR1, NR2B, and NR2A [19]. Furthermore, several studies have demonstrated that EE can counteract cognitive deficits caused by early life stress [20]. Dandi and colleagues reported that EE improved the cognitive decline associated with maternal separation and further reduced corticosterone levels after acute stress exposure [21]. Results of another study demonstrated that exposing mice to EE significantly enhanced hippocampal LTP and cognitive function at the Schaffer's Collateral CA1 synapse [22]. Additionally, researchers indicated that short-term exposure to EE improves working memory, facilitated hippocampal synaptic plasticity, and completely reverses the effects of stress on anxiety behavior [23]. Furthermore, studies have shown that environmental enrichment improves hippocampal-based memory in 60–80 years old adults [24], as well as cognitive dysfunction in developing and aging anesthetics-exposed brains [25].

The aim of this study is to evaluate the effects of postnatal EE on the synaptic plasticity of the hippocampus of noise-stressed prenatal female rats.

## 2. Methods

### 2.1. Animals

Wistar rats provided by the Kashan University of Medical Sciences were housed in a controlled room: humidity (55–60%), temperature (22–24 °C), 12 hours light/dark cycle, and with water and food ad libitum throughout the experiment. Two mature, virgin, female rats were housed together in a cage with a sexually experienced male overnight. If a vaginal plug was observed the next morning, the female rat was considered pregnant and entered the study. Gestation day one was defined as the day the vaginal smear was positive. At gestational day 15, pregnant female Wistar rats were randomly assigned to control and stress groups. After weaning, at postnatal day 21 (P21), half of the prenatally stressed (ST, n = 10) and control offspring (CO, n = 10) were kept in standard cages and the other half were kept in enrichment conditions (SE or CE groups, n = 10 for each).

### 2.2. Prenatal stress protocol

Pregnant rats within the stress group were exposed to 95 dB broadband traffic noise, previously recorded by a recorder (Panasonic RQ-L11) in a high-traffic square, for 2 hours once a day (between 08:00–12:00 am) from the 15th day after mating until the delivery of the pups [26]. A speaker was placed on the upper left of a Plexiglas chamber (25 × 35 × 70 cm) at a distance of 30 cm from the rat cage. A software set the amplitude of the recorded noise at 95 dB (Sonar, Cakewalk, USA), and a sound level meter (Extech Instruments, MA; USA) was used to measure the noise level during the experiments.

### 2.3. Environmental enrichment conditioning

Female offspring at P21 were subjected to EE. Rats were kept in groups of six in EE cages (80 × 40 × 50 cm) with plastic pipes, a steel box, and a wooden ladder. The locations of objects were altered weekly to maintain novelty [27]. The enrichment lasted for one month.

### 2.4. In vivo Electrophysiology

As previously described [28], for electrophysiology recording, the rats were anesthetized with urethane (1.5 g/kg, IP) and fixed in a head holder within a stereotaxic apparatus. (Borj Sanat, Iran). To place the stimulating and recording electrodes in the brain, a drill bit was used to produce two small holes (1 mm diameter) in the skull. A stimulating electrode was initially positioned into the Schaffer's collaterals at the stereotaxic coordinates 4.2 mm posterior to the bregma, 3.4 mm lateral to the midline, and 3.5 mm below the dura surface. The coordinates used for recording electrode were 3.8 mm posterior to the bregma, 2.5 mm lateral to the midline, and 2.8 mm below the dura. The electrodes were prepared from a Teflon-coated stainless-steel wire (A-M Systems, 0.008-inch diameter, USA) exposed only at the tip (tip separation 0.10 mm). The proper location of the electrodes was determined using electrophysiological and stereotaxic indicators. Using a computer software (eProbe, ScienceBeam, Iran), the field excitatory postsynaptic potentials (fEPSPs) were recorded from the CA1 region of the hippocampus in response to stimulation (two sweeps/min at 30-sec intervals) of the ipsilateral to the Schaffer's collateral region. An input-output curve was drawn using a range of stimulus currents when the response was stable. Then, the stimulation intensity was obtained to elicit an fEPSPs amplitude of 60% of the maximum response. Baseline fEPSPs were recorded over a 30-minute period and averaged for comparison with post-tetanus responses. Then, LTP was induced by a 100 Hz high frequency stimulation (HFS) (10 bursts of 10 stimulations, stimulus duration 0.2 ms and interval between bursts of 10 s). After tetanus stimulation, recordings continued for at least 2 hours. Data were considered for the percentage change in amplitude of pre- and post-tetanus recordings.

### 2.5. Serum corticosterone concentration

After electrophysiological recording, blood was sampled from the jugular vein. The plasma was separated into microcentrifuge tubes and stored at −80 ºC until assayed. Plasma corticosterone concentrations were quantified by a radioimmunoassay (RIA) kit (Zellbio, GmbH).

### 2.6. Statistics

All results are shown as means ± SEM. Statistical analysis was performed by a two-way ANOVA followed by Tukey's test. All statistical analyses were completed using the SPSS 20 software, and P values <0.05 were considered statistically significant.

## 3. Results

### 3.1. Effects of prenatal sound stress and postnatal environment enrichment on synaptic plasticity of the Schaffer's collaterals - CA1 pathway

**Figure 1.**
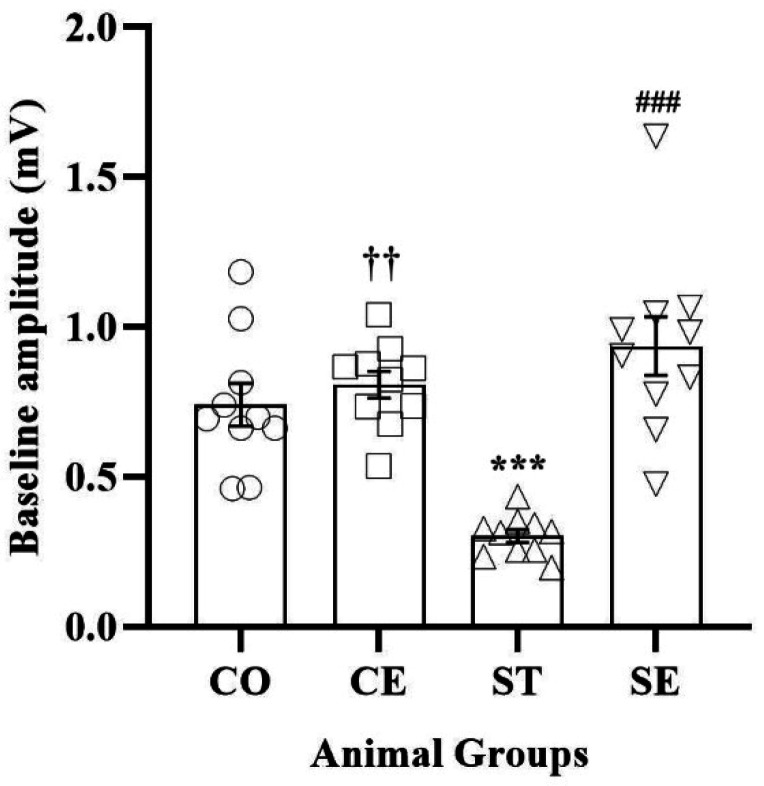
The amplitude of the baseline fEPSPs recorded in the CA1 area of the hippocampus of rats' offspring. Whereas the prenatal noise stress decreased the baseline responses significantly (*** P < 0.001; CO group vs. ST group), the postnatal EE increased fEPSPs more than three times (### P < 0.001; SE group vs. ST group). Also, the postnatal EE increased the baseline responses of the CO animals (†† P < 0.01; CE group vs. CO group).

**Figure 2.**
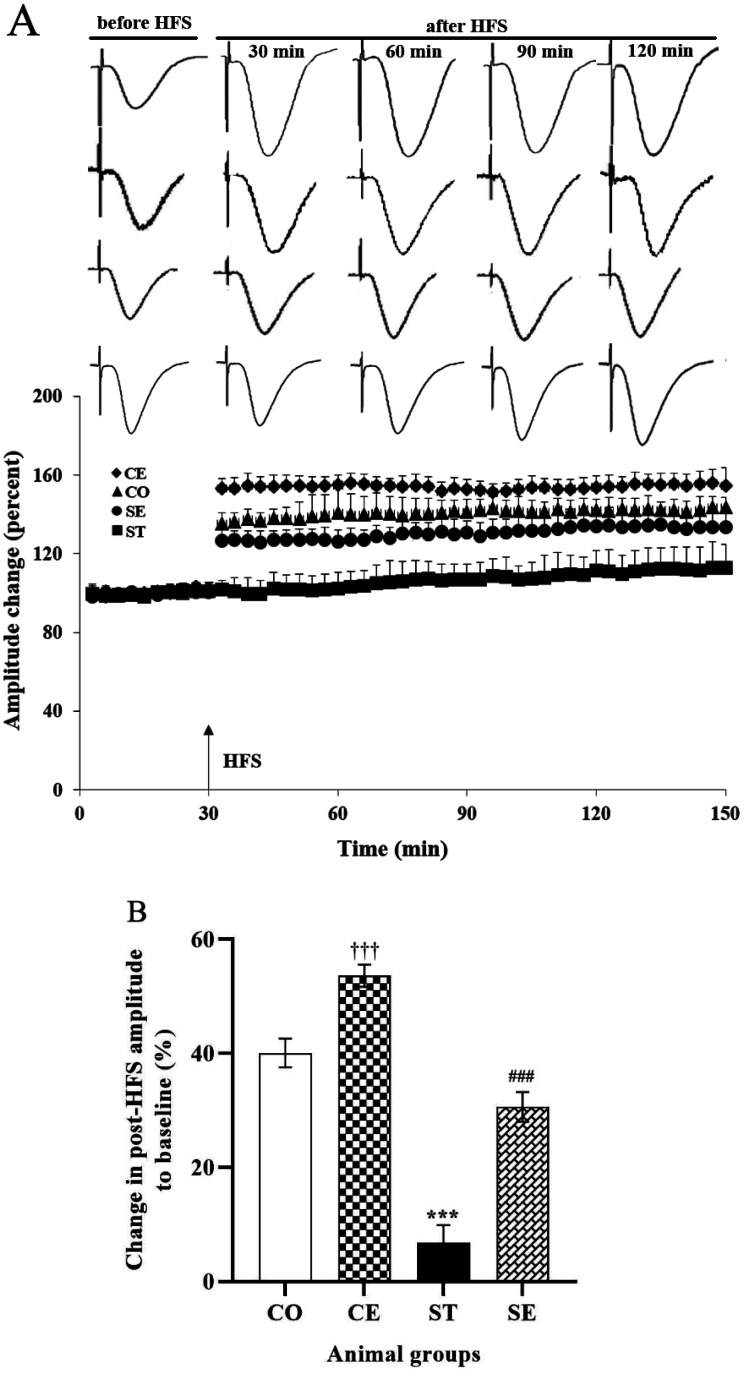
Pre- and post-HFS fEPSPs recorded in the CA1 area of the hippocampus of rats' offspring. While HFS induced LTP in the fEPSPs of the CO rats (*** P < 0.001) it was failed to induced LTP in the fEPSPs of the ST animals (A). The postnatal EE induced LTP about 53% in the controls (††† P < 0.001; CE group vs. CO group) and also by about 30% in the prenatally stressed rats (### P < 0.001; SE group vs. ST group) (B).

Statistical analyses revealed that the interaction of exposure to prenatal sound stress and postnatal environment enrichment changes the amplitude of fEPSPs of female offspring (F_7,5992_ = 19.425; P < 0.001). The mean amplitude size of the baseline response recorded in the CA1 neurons of the rats decreased from 0.74 ± 0.01 mV in the CO rats to 0.30 ± 0.004 mV in the ST rats (P < 0.001) (Figure 1). The EE strikingly reversed the effect of prenatal sound stress on the baseline activity of synapses, where the mean amplitude of the fEPSPs of the SE rats increased to more than three times the fEPSPs of the ST animals (P < 0.001). Additionally, a statistical difference was observed between the mean fEPSPs' amplitude of the CO animals as compared to the CE rats (P < 0.01). In the electrophysiological recordings, basal fEPSPs were evoked by stimulation of the Schaffer's collaterals within the CA1 area of the hippocampus, whereas HFS induced LTP of the excitatory synapses (Figure 2A). There was a statistical difference in the post-HFS potentiation between the CO and ST groups (P < 0.001). On the other hand, LTP induction increased the mean amplitude of the fEPSPs recorded from the CA1 region of CO animals (up to 40%); however, in the ST group, the mean amplitude of the fEPSPs only increased by 6% (Figure 2B). The environmental enrichment deeply affected LTP induction in the CA1 neurons of CE and SE animals, where tetanic stimulation of Schaffer's collaterals induced approximately 53% of LTP in the control group (P < 0.001). Moreover, the EE condition successfully reversed the impairment of the synaptic plasticity for the stressed group, and high-frequency stimulation of the CA3-CA1 pathway induced a significant LTP (~30%) in the fEPSPs recorded for the SE group (P < 0.001). In addition, a two-way ANOVA showed a significant difference between the amplitudes of post-tetanus fEPSPs of the CE and CO animals (P < 0.001).

### 3.2. Effects of prenatal sound stress and postnatal environment enrichment on serum concentration of corticosterone

**Figure 3.**
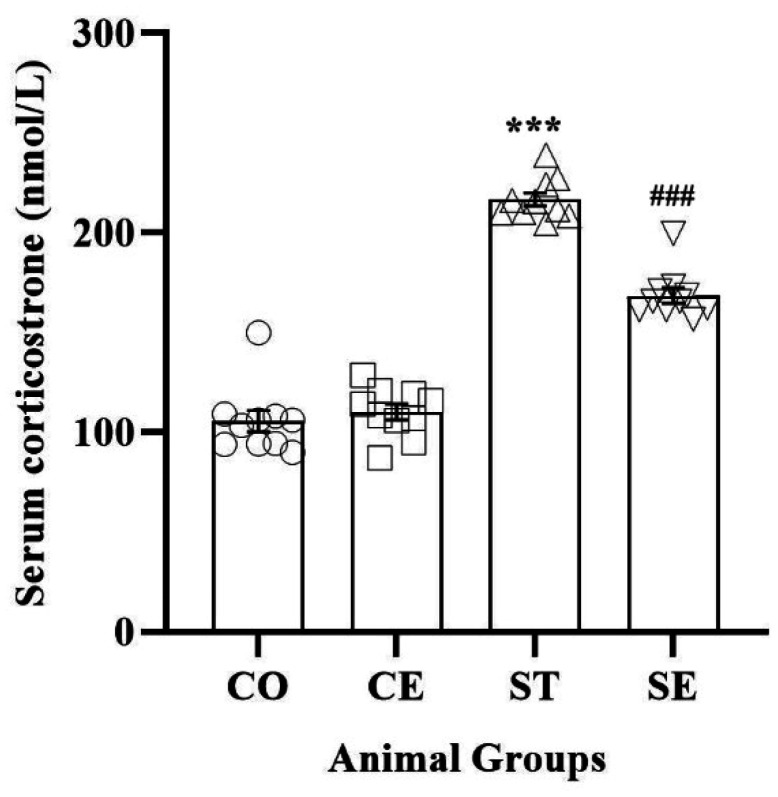
The serum level of corticosterone in the offspring. Although the prenatal noise stress significantly increased corticosterone in the serum of rats' offspring (*** P < 0.001; CO group vs. ST group), the postnatal EE decreased it (### P < 0.001; SE group vs. ST group).

An analysis of variance indicated a substantial difference among the four groups (F_3,36_ = 159.229; P < 0.0001). As shown in Figure 3, two hours of prenatal sound stress significantly increased the serum corticosterone levels to 216.64 ± 3.31 (nmol/L), which was significantly different compared to the CO group (P < 0.001). The statistical analysis revealed that the variation observed between the CO animals (105.51 ± 5.41 nmol/L) and the CE animals (110.16 ± 3.93 nmol/L) was not significant. However, comparing the ST (216.64 ± 3.31 nmol/L) and SE (168.5 ± 3.79 nmol/L) groups showed that postnatal EE decreases the serum level of corticosterone (P < 0.001).

## 4. Discussion

Exposure to stress during pregnancy has long-term effects on offspring because it is crucial for the brain circuitry to form. In agreement with animal data, the results of a study on children whose mothers received exogenous glucocorticoids, faced psychological stress or adverse events during pregnancy, show long-term neurodevelopmental effects [12]. The results of our study demonstrated that exposure to prenatal noise stress between days 14 to 21 of gestation increased the level of serum corticosterone and disrupted the hippocampus-dependent synaptic plasticity of young adult female rats. Furthermore, we found that post-weaning EE positively affects this ameliorated synaptic plasticity and potentiates post-HFS responses of the control rats. Similarly, it has been reported that exposure to PS has a negative effect on LTP induction but enhanced LTD in the hippocampal slices of young rat offspring [29]. Consistent with our results, Barzegar and colleagues reported that exposure to noise stress during pregnancy impaired basal synaptic activity and synaptic plasticity in hippocampal circuits of the rats' male offspring [26].

The exact mechanisms of prenatal stress that affects the synaptic plasticity of the hippocampal circuits in young adult offspring remains to be determined. Although numerous studies have focused on the effects of the HPA-axis and glucocorticoids on learning, memory, and synaptic plasticity, little is known about the downstream mechanism underlying the HPA-axis. In mammals, during embryogenesis, both genetic codes and environmental factors affect the CNS [30], and exposure to any stress chronically affects the programming and development of the offspring's nervous system [31]. Similar to our findings, in one study where rats were exposed to restrainer stress at three weeks of gestation, there was a 50& decrease in the density of the glucocorticoid receptors (GRs) in the hippocampi of their female offspring compared to those of the non-stressed offspring [32]. Mifsud et al. indicated that only exposure to 15 minutes of force swimming stress reduced the number of GRs in the hippocampus of rats [33]. Moreover, it has been reported that either giving glucocorticoid to the fetus [34] or maternal stress [35] leads to the downregulation of GRs in the hippocampus and the development of HPA reactivity; these might be reasons for the alteration in the stress-induced behavior of the offspring [36]. It is well known that prenatal stress can reduce neurogenesis, increase cell death, and enhance hippocampal neurons' oxidative stress in offspring rats [37]. Additionally, prenatal stress reduces the expression and maturation of brain derived neurotrophic factor (BDNF) in the offspring neurons, which is a necessary factor that supports the persistence of long-term memory storage [38]. Furthermore, alterations in GABAergic and glutamatergic systems have been observed in the rats prenatally exposed to stress. There is evidence that PS can reduce glutamate receptor-dependent neuronal synaptic plasticity, thereby leading to impaired learning and memory function [39]. We know that both ionotropic receptors of glutamate α-amino-3-hydroxy-5-methyl-4-isoxazole propionic acid (AMPA) and NMDA are key modulators of hippocampal synaptic plasticity. PS inhibits the expression levels of the NMDA receptor subunits NR2A and NR1 [40] and GluA1-3 subunits of AMPA receptors [41] in the animal's hippocampus. Moreover, PS changes the arrangement of the NMDA receptor subunits that affect function of the NMDA receptors [42]. Feng and colleagues demonstrated that the female offspring of mothers exposed to stress during gestation days 8–20 are more pronounced to PS-induced cognitive dysfunction because of PS-induced changes in the expression of NR2A and NR2B subunits are more prevalent than the male offspring [43]. Furthermore, Adrover et al. revealed that placing rats in a restrainer three times a day for 45 minutes/day during the third week of pregnancy changes glutamate transporters expression, as well as alters glutamate transmission in the offspring brain, which may cause cognitive dysfunction [44]. On one hand, about 10–15 percent of the hippocampal interneurons are GABAergic, and the GABAergic system play critical roles in synaptic plasticity and memory formation [45]. On the other hand, stress has an adverse effect on the GABAergic network structure and function in the hippocampus [46]. Veerawatananan and colleagues showed that maternal forced stress delayed the maturation of the GABAergic system, as well as altered the expression of the GABAA receptor α1 and α5 subunits in the hippocampus of rat pups [47]. Lussiera and Stevens's study revealed that PS reduced the GABAergic cell number and delayed the maturation in the mice hippocampus, which resulted in behavioral dysfunction [48]. Therefore, PS may have long-lasting effects on glutamate and GABA levels and the function of their receptors in the hippocampus, leading to impaired LTP and promotion of LTD in young adult offspring.

We showed that post-weaning EE positively affected both basic synaptic transmission and LTP induction in neural circuits within the CA1 region for both control and PS animals. In line with our results, it has been shown that EE enhances hippocampal LTP and improves learning and memory performance in rats [49]. Additionally, Yang et al. demonstrated that a one-month growth in EE significantly counteracts abnormal alterations in synaptic plasticity induced by PS [50]. It has been shown that EE induces certain hippocampal changes such as increased glial cell number, synaptic density, neurogenesis, and the dendrite branching [51]. For example, EE increases the expression of nerve growth factors including GDNF, NGF, and BDNF in the hippocampus [51]. EE enhances synaptic plasticity and improves various hippocampal-related learning abilities by activating both AMPA and NMDA receptors [52]. On the one hand, Hullinger et al. showed that EE improved learning, memory and hippocampal LTP by increasing mGluR5 activity [53]. On the other hand, Montes et al. indicated that housing in EE for four weeks ameliorated toluene-induced memory impairment in mice and reduced hippocampal GABA levels in these animals [16]. Begenisic and colleagues reported that amelioration of cognitive deficits and synaptic plasticity defects in a mouse model of Down syndrome exposed to EE was associated with decreased hippocampal GABA release [54]. Moreover, EE decreases serum levels of corticosterone and inhibits the anxiety-like behavior provoked immediately after one hour exposure to acute restraint stress [55]. Dandi et al. reported that EE protects against cognitive dysfunction caused by maternal separation and decreases corticosterone levels [56].

## 5. Conclusions

In conclusion, we showed that postnatal EE restored the stress induced impairment of synaptic plasticity within the CA1 area of rats' female offspring. It appears that the reduction of the stress hormone corticosterone and alterations in the function and structure of the hippocampus, especially the balance between excitatory and inhibitory transmission, are possible mechanisms through which EE favors synaptic plasticity.
